# Localized surface plasmon enhanced photothermal conversion in Bi_2_Se_3_ topological insulator nanoflowers

**DOI:** 10.1038/srep25884

**Published:** 2016-05-12

**Authors:** Jia Guozhi, Wang Peng, Zhang Yanbang, Chang Kai

**Affiliations:** 1 Tianjin Chengjian University, Tianjin 300384, PR China; 2 Institute of Semiconductors, Chinese Academy of Sciences, P.O. Box 912, Beijing 100083, PR China

## Abstract

Localized surface plasmons (LSP), the confined collective excitations of electrons in noble metal and doped semiconductor nanostructures, enhance greatly local electric field near the surface of the nanostructures and result in strong optical response. LSPs of ordinary massive electrons have been investigated for a long time and were used as basic ingredient of plasmonics and metamaterials. LSPs of massless Dirac electrons, which could result in novel tunable plasmonic metamaterials in the terahertz and infrared frequency regime, are relatively unexplored. Here we report for first time the observation of LSPs in Bi_2_Se_3_ topological insulator hierarchical nanoflowers, which are consisted of a large number of Bi_2_Se_3_ nanocrystals. The existence of LSPs can be demonstrated by surface enhanced Raman scattering and absorbance spectra ranging from ultraviolet to near-infrared. LSPs produce an enhanced photothermal effect stimulated by near-infrared laser. The excellent photothermal conversion effect can be ascribed to the existence of topological surface states, and provides us a new way for practical application of topological insulators in nanoscale heat source and cancer therapy.

Surface-bound collective excitations of free carriers in noble metals, doped semiconductors or graphene can be excited by light in resonance with the driving electromagnetic field^1^,^2^,^3^. Due to enhanced near-surface electric fields, so-called localized surface plasmon resonances (LSPRs), the nanocrystals show intense light absorption and scattering. The strongly confined LSPRs have been the basic ingredients in subwavelength microscopy, near-field lithography, and nanophotonics. Their properties are promising for potential applications, ranging from photovoltaics^4^ to bio-imaging^5^,^6^ and photothermal therapy^7^.

Recently, plasmons of massless Dirac electrons have been observed in graphene, a purely two-dimensional (2D) electron systems^3^. The oscillation frequency of plasmons can be tuned by electric gating ranging from the terahertz to infrared frequency regimes. Massless Dirac fermions also occur in the 2D surface states of three-dimensional topological insulators (TIs) existing in the bulk gap. Topological insulators are a new class of quantum matters with an insulating bulk and metallic surface states, in which electrons behave like massless Dirac fermions. The surface states are protected by time-reversal symmetry and exhibit spin-momentum locking, i.e., the chirality, which forbid backscattering processes. In recent years, TIs have stimulated intensive interest, not only because of their unique electronic structures, but also for their potential applications ranging from spintronics^8^, photocatalysis^9^, and thermoelectric transport^10^ to quantum computing^11^. TI surface states also appear as a purely 2D Dirac fermion system like graphene^12^, but without the need to physically implement an atomically thin monolayer. Very recently, plasmons in topological insulator surface states are observed experimentally in Bi_2_Se_3_ periodically arranged microribbon arrays^13^, and the electric field **E** of electromagnetic wave is required to perpendicular to the microribbon arrays. This configuration is made to satisfy the requirement of the dispersion of surface states and the spin-momentum locking, which prevent the momentum conversation in photon absorption. The periodically arranged ribbon arrays provide an in-plane extra momentum, which makes it possible to generate plasmonic excitation by electromagnetic radiation. A fundamental question of considerable importance is how the surface plasmons in three-dimensional(3D) TIs evolve at nanoscale. Due to strong quantum confinement, one can expect that the topological surface states are quantized^10^ and the energy-momentum conservation will be relaxed, making it more easy to excite the collective excitation, i.e., localized surface plasmons (LSPs) in TI nanostructures.

Recent studies show that high density free carriers result in localized surface plasmons in the near- or mid-infrared frequency regime in highly self-doped Cu_2x_S semiconductor nanostructures^2^. The oscillation of LSP can be enhanced through resonance with the driving electromagnetic field, resulting in surface-enhanced Raman scattering, which depend on the generation of LSPs at interfaces dielectric core and noble metal shell. In contrast to this hybridized dielectric/metal nanostructures, TI nanostructures posses exotic metallic surface states, therefore, one can expect that the metallic surface states can substantially enhance local fields near the surface and affect the optical property of TI nanostructures. Surface-enhanced Raman scattering can offer us an efficient and powerful tool to probe the TI surface states.

Here, we report for first time the LSPs in Bi_2_Se_3_ TI nanostructures and photothermal conversion in such systems. Effect of quantum confinement on the exotic surface states can effectively reduce the bulk contribution and more easily to open the gap and probe the exotic surface states^14^,^15^. Superior performance can be predicted in zero-dimensional (0D) nanostructure due to enhanced surface-to-volume ratios. Electrons in TI nanostructures can interact with electromagnetic radiation due to the relaxation of the momentum conservation, resulting in the LSP excitations. Utilizing enhanced local field near the surface of TI nanostructures, we find an excellent photothermal conversion in TI nanostructures, which is comparable with or even better than that in conventional semiconductor nanostructures coated with noble metal shell. Our work demonstrates the TI nanostructures could be used in nanoscale heat sources and cancer therapy, paves a completely new way toward practical applications of TIs.

## Results

**Figure 1:**
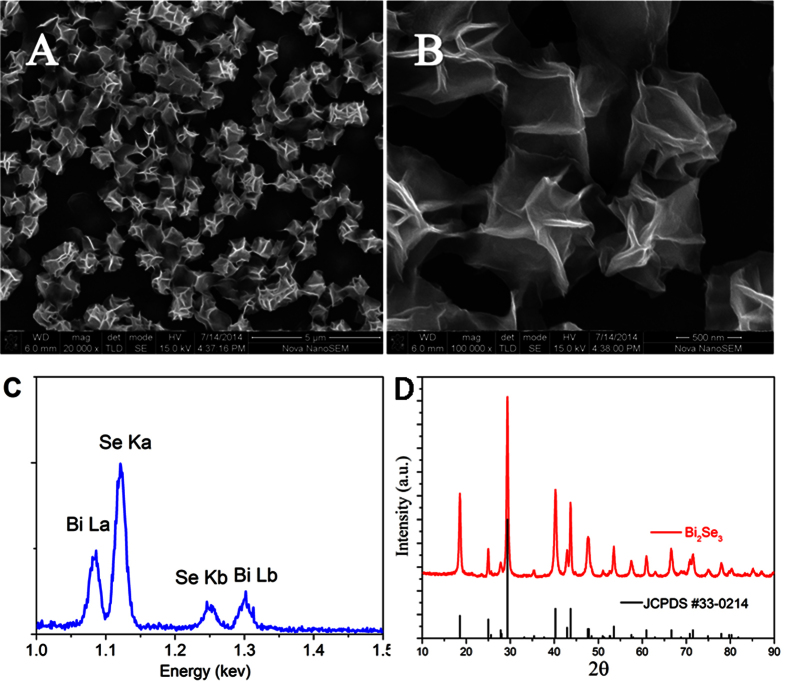
Morphology and structure of Bi_2_Se_3_ nanomaterials. SEM images of hierarchical Bi_2_Se_3_ NF samples (**A,B**). (**C**) The energy-dispersive X-ray spectroscopy (EDS) of Bi_2_Se_3_ NFs. **(D)** XRD pattern of the sample of Bi_2_Se_3_.

The samples of Bi_2_Se_3_ nanoflowers (NFs), composed of a large number of Bi_2_Se_3_ nanocrystals (NCs), are prepared in solution-based process assisted by microwave irradiation^16^. The scanning electron microscopy (SEM) images clearly illustrate that the as-synthesized products are consisted of well-defined NFs with narrow size distribution and a typical diameter of 800 nm (see Fig. 1A). High-resolution SEM images show that the NFs with smooth surfaces are composed of many leaf-like ultrathin membranes (Fig. 1B), which are consisted of numerous TI nanocrystals. The compositions of the sample determined by energy dispersive spectroscopy (EDS) was shown in Fig. 1C, confirming the presence of Se and Bi atoms, which demonstrated that Se NCs can serve as soft templates for preparing Bi_2_Se_3_ NFs^17^,^18^. X-Ray diffraction (XRD) patterns are used to determine the composition and structure of the sample synthesized by two-step method with the assistance of PVP as shown in Fig. 1D. The main diffraction peaks can be readily indexed into the rhombohedral phase of Bi_2_Se_3_, which match well with the reported value (JCPDS Card No. 33-0214). The well-defined peaks in XRD pattern indicate the forming of high quality of NCs.

**Figure 2:**
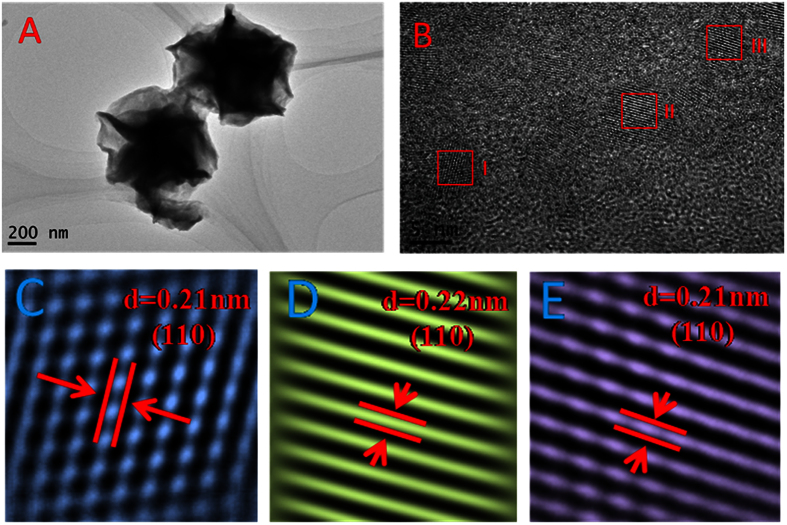
Bi_2_Se_3_ nanocrystals forming and crystalline structure. (**A**) Typical TEM image of Bi_2_Se_3_ NFs. **(B)** The TEM image showing the high quality crystalline structure. (**D–F)** Inverse transforms of contrast-enhanced FFTs of the marked areas in Fig. 2B.

Size distribution and crystal structure have been evaluated by HRTEM, which provides us further information about the details of the hierarchical NF structure. Figure 2A shows the NFs structures, which is in good agreement with the SEM measurements. In order to check the quality of the sample, the HRTEM images of three selected regions are shown in Fig. 2B, respectively. HRTEM images are digitally processed using a 2D Fourier transform scheme, and inverse transformed to obtain the 2D Fourier-transform filtered lattice fringes (see Fig. 1C–E) to precisely measure the lattice spacing. It can be clearly seen that the fringes corresponds to the red box I, II, and III in Fig. 2B are almost perfect single crystal with 0.21 nm lattice spacing between atoms, which is consistent with the lattice constant in (110) planes of Bi_2_Se_3_. The SEM and HRTEM images indicate that the Bi_2_Se_3_ NF sample is grown along the c-axis direction and an ultrathin film, and clearly show each NF is composed of Bi_2_Se_3_ NCs with small diameter (~5 nm).

These unique NCs may exist some important physical performances due to the strong 3D confinement. First it is necessary to analyze the forming process of Bi_2_Se_3_ NCs. During the process of sample preparing, the two-step synthesis are crucial to the fabrication of Bi_2_Se_3_ NFs. Se NCs were firstly formed with the protection of surfactant PVP. The size and morphology of Se NCs strongly depend on the experimental conditions, such as dosage of PVP, reaction time, microwave power, and pH value etc. The Se nanoparticle powder cannot be obtained due to extremely small size and PVP coverage at the surface of Se nanoparticle. Se nanoparticles play a dual role, on the one hand, it can react with Bi ion and form Bi_2_Se_3_ NCs, and on the other hand, it can be as the nucleation centers for growth of Bi_2_Se_3_ NFs. The validity of the two-step synthesis can be demonstrated by the results of XRD and TEM. In addition, PVP plays a critical role during the forming process of Bi_2_Se_3_ NCs and controlling of morphology. It can prevent aggregation of particles during the forming of the NCs as a stabilizing agent, and resulting in a uniform colloidal dispersion. PVP is also can promote reduction onto specific crystal faces while preventing reduction onto other crystallographic planes^19^. In addition, the aqueous dispersion of Bi_2_Se_3_ NFs has high stability due to the presence of the PVP ligands on the surface of hierarchical Bi_2_Se_3_ architectures NCs. In addition, layer structured characteristics of Bi_2_Se_3_ material can determine the growth behavior, and lead to the few quintuple layer formed.

**Figure 3:**
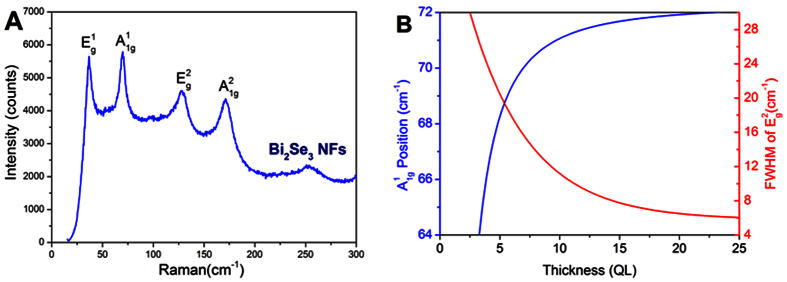
Raman spectra enhanced by localized surface plasmon resonances in Bi_2_Se_3_ NCs. **(A)** Raman spectrum of Bi_2_Se_3_ NFs in the range of 0–300 cm^−1^. **(B)** The peak position of 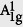 mode and broadening of 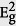 mode as s function of the sample thickness.

Raman spectrum are efficient tools to investigate the optical property and shape characteristics of Bi_2_Se_3_ NFs^20^,^21^,^22^. Raman spectroscopy with a 632 nm excitation laser (15 mW) is also performed to investigate the electron states in the samples in a backscattering configuration. Figure 3(A) shows typical Raman spectra obtained from as-prepared samples. It can clearly be seen that four main Raman peaks assigned to the vibrational modes 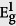
(~36 cm^−1^), 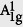
(~72 cm^−1^), 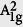
(~105 cm^−1^), and 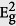
(~172 cm^−1^), respectively. It can see clearly that the lowest frequency 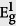 mode is very strong, which can be ascribed to the LSP enhanced Raman signal^21^. Layer structured Bi_2_Se_3_ has the rhombohedral crystal structure, composed of three quintuple layers stacked together by the Van der Waals forces. The size of material can strongly affects the shift of the peak 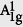 corresponding to the out-of-plane phonon mode and the broadening of the peak of the out-plane mode 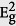 in the Raman spectrum for Bi_2_Se_3_ NCs. By compared with bulk mode of 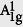, a pronounced red shift can be observed. Because the out-of-plane vibration modes of the Se and Bi atoms are very sensitive to the thickness of Bi_2_Se_3_ NF sample, the size of NCs can be estimated by the peak shift of 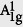 mode and Raman band broadening of 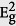 mode according to the empirical formula^21^. Figure 3B displays the relationship between the size and the peak position of 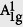 mode and broadening of 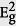 mode. By comparison the 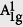 mode between theoretical and experimental results, the sizes of Bi_2_Se_3_ NCs are about 5 nm, which agree well with the TEM results.

Figure 3B shows the absorbance results of Bi_2_Se_3_ NFs. No absorption peak is observed in the near- or mid-infrared band for bulk Bi_2_Se_3_ due to the extremely narrow band gap. It can been clearly seen that the characteristic peaks appear in the absorbance spectrum of the Bi_2_Se_3_ NCs. The remarkably well-defined peaks in the absorbance spectrum correspond to the free-carrier absorption in Bi_2_Se_3_ NFs. The LSPR modes in the Bi_2_Se_3_ NFs are completely different from that in noble metals and self-doped semiconductor nanocrystals^2^. Generally, the density of free carriers plays an important role for the forming of LSPs in NCs. The LSPs of the perfectly stoichiometric Bi_2_Se_3_ depend on the two main factors. As materials are exposed to ambient air, the surface of Bi_2_Se_3_ NCs may adsorb hydroxide oxide from air, which can result in the reversed doping effect and band bending, which may originate from a hydroxide oxide process^23^. This can lead to a n-type surface doping for Bi_2_Se_3_ NCs. In addition, the surface/volume ratio of NCs is inversely proportional to the size of NCs, resulting in increasing of the density of free carriers. High-density free carriers can occupy the topological surface states and cause resonance with the driving electromagnetic field, and consequently lead to LSP enhanced surface Raman scattering and absorbance spectrum.

## Discussion

**Figure 4:**
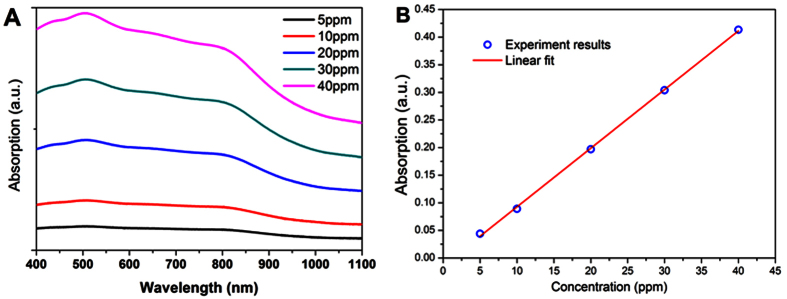
Absorbance spectra enhanced by localized surface plasmon. (**A**) Absorption spectrum of the aqueous dispersion of nanoflowers with different concentrations (5, 10, 20, 30, and 40 ppm). (**B**). The absorbance spectrum as a function of the concentration of Bi_2_Se_3_ NFs in water.

**Figure 5:**
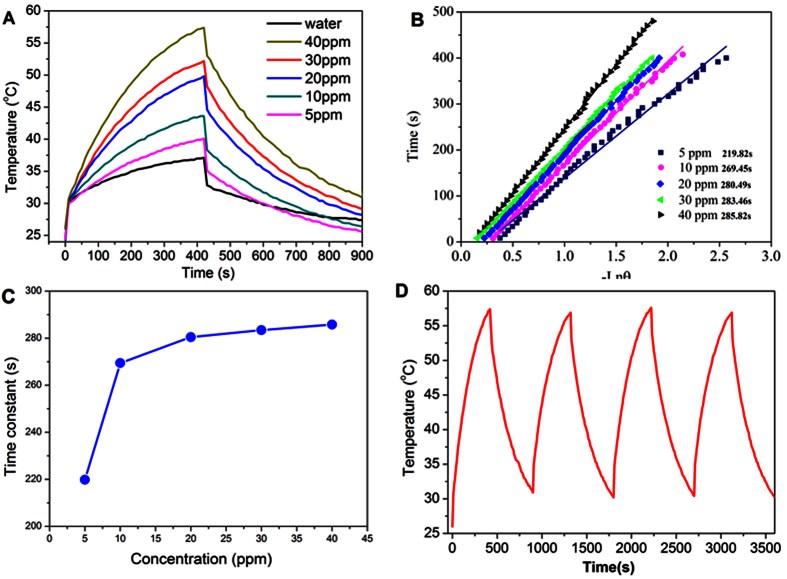
Photothermal conversion of Bi_2_Se_3_ NFs. (**A**) Photothermal conversion effect of pure water and the aqueous dispersion of with different concentrations(5, 10, 20, 30, and 40 ppm) as a function of irradiation time (7 min) using the NIR laser shining (808 nm, 1.6 W) for 7 min, and shut off then. (**B**) Time constant for heat transfer obtained by fitting. (**C**) The relationship between time constant and concentration obtained in Fig. 5(B,D) Temperature evolution of the sample over four laser ON/OFF cycles.

Currently, photothermal conversion have been a powerful way to analyze the LSP in nanomaterials^24^,^25^,^26^. Next, we will study the photothermal effect in such Bi_2_Se_3_ NFs. Considering that the photothemal conversion performance of nanomaterials largely depends on the absorption characteristic at the NIR band^27^. We analysed the UV-vis-NIR absorbance spectra of Bi_2_Se_3_ NCs with different concentrations in water solution. Figure 4A shows the UV-vis-NIR absorbance spectra of Bi_2_Se_3_ NFs dispersed in water at room temperature. It can be clearly seen that there are two broadened absorbance peaks cantered at 500 nm and 800 nm. The peak near 808 nm is caused by the localized surface plasmon resonance (LSPR), which could play a dominant role for the photothermal effect in Bi_2_Se_3_ NFs. The absorbance increases linearly as the concentration of Bi_2_Se_3_ NFs in water (see Fig. 4B). Importantly, we would like to emphasize that this linear behavior indicates that Bi_2_Se_3_ NFs in water solution are highly stable and uniformly distributed. The observed peak near 808 nm is in good agreement with recent theoretical work by Vargas *et al.* for Bi_2_Se_3_ NCs based on tight-binding theory^14^. As the sizes of Bi_2_Se_3_ NCs decrease, the LSPR peak appear, and approach the near infrared band, which could be very promising for application of the photothermal conversion in cancer therapy. 808 nm NIR laser was delivered through a quartz cuvette containing aqueous dispersion NFs to measure the photothermal conversion performance of hydrophilic Bi_2_Se_3_ NFs. Surprisingly, temperatures of solution exhibit remarkable increase within 4 mins under irradiation of 808 nm laser (1.6 W). The temperature of Bi_2_Se_3_ suspension rose rapidly to 66.5 °C within 4 min at the density n = 40 ppm, but only 40 °C for n = 5 ppm, as shown in Fig. 5A. The efficiency of the photothermal conversion increases with increasing the densities of Bi_2_Se_3_ NFs in water solution. These results clearly show that Bi_2_Se_3_ NFs could potentially act as the efficient photothermal conversion agent. The photothermal conversion efficiency of nanopariticales was determined based on the macroscopic model.


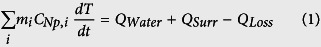


where *m* and 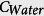 are the mass and heat capacity of water and *T* is the solution temperature. The photothermal energy from the Bi_2_Se_3_ NFs Q_Np_ can be written as


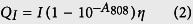


where *I* is the laser power, 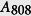 is the absorbance at the excitation wavelength of laser, and 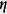 is the photothermal conversation efficiency. The heat lost to the surroundings by the cuvette walls 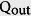 was given as.


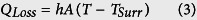


where 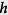 his heat transfer coefficient, 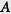
is the surface area of the container, 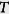
and 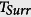 is ambient temperature of the surroundings. The temperature profile after the laser is turned on/turn off can be obtained by solution of the equation (1). Therefore, the photothermal conversion efficiency can be determined as


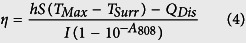


The water-dispersed Bi_2_Se_3_ NFs with a photothermal conversion coefficient of 30.06% were synthesized by a two-step reaction. The thermal equilibrium time constant can effectively evaluate the heat storage capacity, and can be determined by heat transfer equation^28^,^29^. The thermal equilibrium time constants of the aqueous dispersion of NFs with different concentrations were obtained for thermal equilibration with the surroundings via conductive and irradiative heat transfer. Figure 5B shows a time constant for heat transfer time determined as the negative reciprocal slope of ln(θ) vs. t using temperature versus time data recorded during cooling of the solution (see Fig. 5A). Therefore, the thermal equilibrium time constant of the samples are calculated to be 219.82, 269.45, 280.49, 283.46 and 285.82 s for the concentrations 5, 10, 20, 30, and 40 ppm, respectively. It can clearly be seen that the heat transfer time increase with increasing of the Bi_2_Se_3_ NFs concentrations. The specific thermal equilibrium time constant can be analyzed as follow^29^,


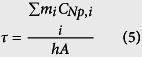


As shown in Fig. 5C, the thermal equilibrium time constant for the different concentrations increase dramatically as the concentration of sample increases to 10 ppm, and then exhibits a saturation as the concentration of Bi_2_Se_3_ NFs further increases to 20 ppm, which can be attributed to the equilibrium of heat generation and transfer to environment. This result proves the excellent heat storage capacity behavior of the Bi_2_Se_3_ NFs. Excellent cycling stability is another important characteristic for high-performance photothermal conversion agent. Figure 5D reveals the temperature elevation cycle performance of Bi_2_Se_3_ NFs samples over four laser ON/OFF cycles of 808 nm NIR laser irradiation. No significant decrease for the temperature elevation was observed for our samples, which indicating excellent thermal stability of the Bi_2_Se_3_ NFs. From our experiment, we demonstrate that Bi_2_Se_3_ NCs existing in NFs are dominant heat sources. It is worthy to point out that the LSP, heat storage capacity and heat stability of Bi_2_Se_3_ NCs play crucial roles for excellent photothermal conversion in Bi_2_Se_3_ NFs.

**Figure 6:**
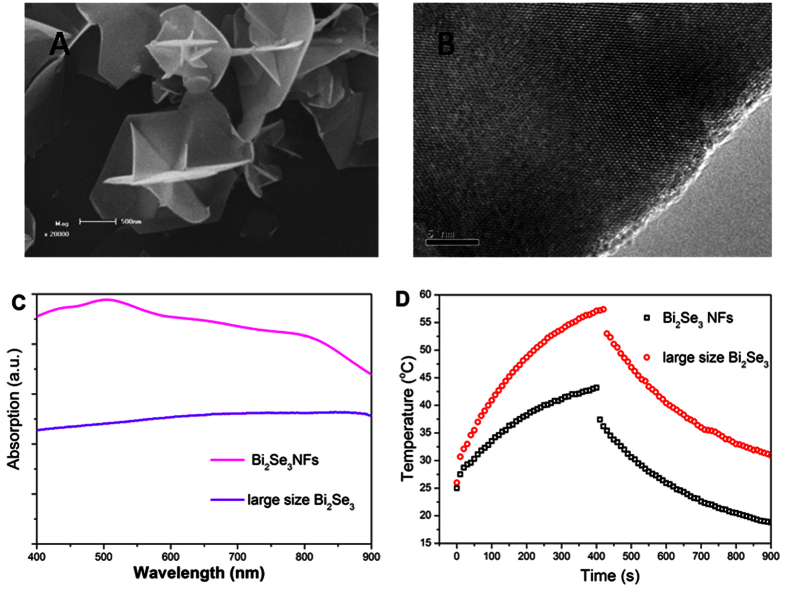
Comparison of absorption and photothermal conversion between Bi_2_Se_3_ NFs and large size single crystal Bi_2_Se_3_. (**A**) SEM images of Bi_2_Se_3_ NFs consisted of large size NCs (LNF). (**B**) TEM image of large size single crystals Bi_2_Se_3_ samples. (**C**) Absorption spectrum of the Bi_2_Se_3_ NFs with small NCs and large size single crystals Bi_2_Se_3_. (**D**) Photothermal effect the aqueous dispersion of NFs and large size single crystals as a function of irradiation time (7 min).

In order to clearly understand the effect of LSP on the photothermal conversion of Bi_2_Se_3_ NFs, we compare the absorption property and photothermal conversion of small size Bi_2_Se_3_ NCs with the sample containing large size Bi_2_Se_3_ NCs. SEM (Fig. 6A) and TEM (Fig. 6B) images show that as-synthesized large size Bi_2_Se_3_ NCs have an average diameter of 1 μm. The absorption spectrum (Fig. 6C) of the sample containing large size Bi_2_Se_3_ NCs does not show significant absorption enhancement for laser irradiation whose frequency tuned from 400 nm to 900 nm. In a further photothermal conversion experiments, it can be clearly seen that the aqueous dispersion of the small size Bi_2_Se_3_ NCs exhibits a higher efficiency of photothermal conversion, i.e., the higher temperature, under the same irradiation time, laser power, and solution concentration. This difference is caused by the different carrier densities in the samples containing small and large Bi_2_Se_3_ NCs due to different surface/volume ratio. The sample containing small Bi_2_Se_3_ NCs possesses larger surface/volume ratio, which makes it possible to absorb and gain more electrons from adsorption hydroxide oxide from air^14^. The appearance of a plasma absorption peak provided direct evidence of the increasing amount of free electron associated with surface/volume ratio. The surface to volume ratio of NCs can be efficiently enhanced by decreasing the size of NCs. which can critically shift the LSP peak to the NIR band and enhance the absorption. In addition, Bi_2_Se_3_, with an extremely narrow bandgap, the absorbed photon energy from 808 nm laser is well above the bandgap, which make it possible to lost the energy gained from photons as heat through electron-phonon scattering and subsequent phonon emission processes during relaxation of the photo-excited carriers to the band edges^30^. Furthermore, Bi_2_Se_3_ hierarchical NFs were composed of 0-dimensional confinement NCs, resulting in the gap of Bi_2_Se_3_ opened, which can effectively inhibit the carriers recombining^14^.

## Conclusion

In summary, we demonstrate experimentally that hydrophilic Bi_2_Se_3_ NFs are novel photothermal agents prepared by a microwave assisted technique. The aqueous dispersion of the Bi_2_Se_3_ NFs (40 ppm) exhibits an enhancing absorbance under the irradiation of 808 nm laser. The excellent photothermal conversion effect in such system could pave a new way for practical application of topological insulator in nanoscale heat source and cancer therapy. The mechanism of excellent photothermal conversion effect in the Bi_2_Se_3_ NFs can be ascribed to the enhanced absorption due to the LSPs caused by the surface states in Bi_2_Se_3_ NFs.

## Methods

### Bismuth selenide NFs assembled from nanosheets

The samples were synthesized using solution-based process assisted by microwave irradiation. In a typical procedure for synthesizing samples, the PVP was used as surfactant and ethylene glycol as solvent. In brief, 0.3068 g PVP, 0.3068 g Na_2_SeO_3_·5H_2_O, 0.2495 g NaOH, and 30 mL ethylene glycol, were added in a 150 mL three-neck round bottom flask and vigorously stirred at room temperature for 30 min. The Se nanocrystals were obtained after 5 min microwave irradiation of the precursor solution. Bi precursor solution was prepared by dissolving 0.0783 g PVP, and 0.3773 g Bi(NO_3_)_3_·5H_2_O in 3 mL ethylene glycol. The temperature of Bi precursor solution was increased to 120 °C and keeping constant stirring. Bi precursor solution was rapidly injected into Se nanocrystals solution. The mixture solution was reacted under 400 W microwave power at 120 °C for 20 min. The solution was allowed to cool down to room temperature naturally after the reaction was stopped. The black precipitated powder was collected and washed several times carefully. The samples were finally obtained after natural drying.

### Synthesis of the large size bismuth selenide single crystal NFs

0.2424 g Bi(NO_3_)_3_·5H_2_O and 50 mL ethylene glycol were added in a 150 mL three-neck round bottom flask and stirred at room temperature for 30 min. Then, 0.1297 g Na_2_SeO_3_ and 0.5 g NaOH were added into flask, and elevating temperature to 180 °C under nitrogen environment and keeping constant stirring. The reaction was stopped after 80 min and the solution was allowed to cool down to room temperature naturally. The black precipitated powder was collected and washed several times using acetone and water, respectively. The samples were finally obtained after drying under a vacuum at 50 °C for several hours.

### Morphology and structure characteristics of samples

The crystalline structures of the samples were investigated by X-ray diffraction (Rigaku-D/MAX-2550PC, Cu Ka radiation, λ = 1.54056 Å). The morphology of the obtained samples was assessed on a field emission scanning electron microscope (FESEM, FEI Quanta 200F) and with transmission electron microscopy (TEM, FEI Tecnai G2 S-Twin) with an operating voltage of 300 kV. Scanning transmission electron microscopy X-ray energy dispersive spectrometry (STEM-XEDS) was also performed on an FEI Tecnai G2 S-Twin transmission electron microscope, equipped for energy dispersive X-ray spectroscopy in the STEM mode. The elemental composition was investigated with a 1 nm probe size and 20 cm camera length.

### Optical prosperties and photothermal measurements

Raman spectroscopy was performed using a 632.8 nm laser with an incident power of 0.5 mW. UV-Vis absorption spectra were obtained using a Perkin Lambda UV-Vis-near-infrared spectrophotometer. For measuring the photothermal conversion performance of Bi_2_Se_3_ samples, 808 nm NIR laser was delivered through a quartz cuvette containing aqueous dispersion (1.0 mL) of samples with the same concentrations, and the light source was an external adjustable power 808 nm semiconductor laser device with a 5 mm diameter laser module. The output power was 1.6 W for a spot size of ~0.6 cm^2^. A thermocouple with an accuracy of ± 0.1 °C was inserted into the aqueous dispersion of samples perpendicular to the path of the laser.

## Additional Information

**How to cite this article**: Guozhi, J. *et al.* Localized surface plasmon enhanced photothermal conversion in Bi_2_Se_3_ topological insulator nanoflowers. *Sci. Rep.* **6**, 25884; doi: 10.1038/srep25884 (2016).
